# Loss of the Urothelial Differentiation Marker FOXA1 Is Associated with High Grade, Late Stage Bladder Cancer and Increased Tumor Proliferation

**DOI:** 10.1371/journal.pone.0036669

**Published:** 2012-05-10

**Authors:** David J. DeGraff, Peter E. Clark, Justin M. Cates, Hironobu Yamashita, Victoria L. Robinson, Xiuping Yu, Mark E. Smolkin, Sam S. Chang, Michael S. Cookson, Mary K. Herrick, Shahrokh F. Shariat, Gary D. Steinberg, Henry F. Frierson, Xue-Ru Wu, Dan Theodorescu, Robert J. Matusik

**Affiliations:** 1 Department of Urologic Surgery, Vanderbilt University Medical Center, Nashville, Tennessee, United States of America; 2 Department of Pathology, Vanderbilt University Medical Center, Nashville, Tennessee, United States of America; 3 Department of Surgery, University of Chicago, Chicago, Illinois, United States of America; 4 Department of Public Health Sciences, University of Virginia, Charlottesville, Virginia, United States of America; 5 Department of Urology, Weill Cornell Medical College, New York, New York, United States of America; 6 Department of Pathology, University of Virginia, Charlottesville, Virginia, United States of America; 7 Deparments of Urology and Pathology, New York University, New York, New York, United States of America; 8 University of Colorado Comprehensive Cancer Center, Aurora, Colorado, United States of America; 9 Department of Cancer Biology, Vanderbilt University Medical Center, Nashville, Tennessee, United States of America; 10 Department of Cell and Developmental Biology, Vanderbilt University Medical Center, Nashville, Tennessee, United States of America; University of Central Florida, United States of America

## Abstract

Approximately 50% of patients with muscle-invasive bladder cancer (MIBC) develop metastatic disease, which is almost invariably lethal. However, our understanding of pathways that drive aggressive behavior of MIBC is incomplete. Members of the FOXA subfamily of transcription factors are implicated in normal urogenital development and urologic malignancies. FOXA proteins are implicated in normal urothelial differentiation, but their role in bladder cancer is unknown. We examined FOXA expression in commonly used *in vitro* models of bladder cancer and in human bladder cancer specimens, and used a novel *in vivo* tissue recombination system to determine the functional significance of FOXA1 expression in bladder cancer. Logistic regression analysis showed decreased FOXA1 expression is associated with increasing tumor stage (p<0.001), and loss of FOXA1 is associated with high histologic grade (p<0.001). Also, we found that bladder urothelium that has undergone keratinizing squamous metaplasia, a precursor to the development of squamous cell carcinoma (SCC) exhibited loss of FOXA1 expression. Furthermore, 81% of cases of SCC of the bladder were negative for FOXA1 staining compared to only 40% of urothelial cell carcinomas. In addition, we showed that a subpopulation of FOXA1 negative urothelial tumor cells are highly proliferative. Knockdown of FOXA1 in RT4 bladder cancer cells resulted in increased expression of UPK1B, UPK2, UPK3A, and UPK3B, decreased E-cadherin expression and significantly increased cell proliferation, while overexpression of FOXA1 in T24 cells increased E-cadherin expression and significantly decreased cell growth and invasion. *In vivo* recombination of bladder cancer cells engineered to exhibit reduced FOXA1 expression with embryonic rat bladder mesenchyme and subsequent renal capsule engraftment resulted in enhanced tumor proliferation. These findings provide the first evidence linking loss of FOXA1 expression with histological subtypes of MIBC and urothelial cell proliferation, and suggest an important role for FOXA1 in the malignant phenotype of MIBC.

## Introduction

It is estimated that in 2011 over 69,250 people in the United States will be diagnosed with carcinoma of the urinary bladder [1]. More than 90% of bladder cancers are histopathologically classified as urothelial cell carcinomas (UCC), while adenocarcinomas, squamous cell carcinomas (SCC) and small cell carcinomas represent less common histological variants. Most patients present with non-invasive disease, but often develop recurrence, sometimes with progression to stromal invasion. Thus, vigilant surveillance of these patients by periodic cystoscopy and urine cytology is required following tumor treatment. Clinical management for patients with Ta or Tis disease is therefore extraordinarily expensive [1]. On the other hand, clinical intervention following the diagnosis of muscle invasive bladder cancer (MIBC; tumor stage≥T2) typically entails radical cystectomy. Despite aggressive surgical intervention, approximately 50% of patients undergoing radical cystectomy will experience disease recurrence, usually in the form of metastatic disease. The development of metastatic disease is almost invariably lethal, and it is estimated in 2011 that over 14,990 individuals in the United States will perish from metastatic bladder cancer in the United States [1].

Relative to other malignancies, bladder cancer is severely understudied and underfunded. Given the high cost of surveillance and high mortality rate in patients with advanced disease, increased efforts to define the biological pathways critical to such pressing clinical problems of tumor recurrence, as well as progression to muscle invasion, and/or distant metastasis are needed. One approach is to identify those pathways that influence normal differentiation that are perturbed during tumor initiation and progression. One group of proteins that appears to play an important role in the development and control of tissue-specific expression in bladder urothelium consists of select members of the Forkhead Box (FOX) family of transcription factors. Several recent reports have implicated a central role for one member of this family, FOXA1, in urothelial differentiation [2], [3], [4], [5], [6], [7]. While FOXA1 is expressed in normal adult murine and human urothelium, the extent of FOXA family member expression in bladder carcinoma is unknown. Since other FOX proteins have been implicated in the development and progression of a variety of malignancies [8], we initiated a study to interrogate FOXA family member expression in human bladder cancer cell lines and in human tumor samples, as well as to determine the functional role of FOXA1 in a tissue recombination model of urothelial tumor cell biology.

## Materials and Methods

### Ethics Statement

De-identified human bladder tissue samples were obtained from the Vanderbilt Tissue Acquisition Core via the Department of Pathology in accordance with Vanderbilt IRB protocols. Tissue microarrays were created as previously described [9] from de-identified human bladder tissue obtained from the University of Virginia, and was used in accordance with University of Virginia IRB protocols. All patients signed informed consent approving the use of their tissues for unspecified research purposes. All experiments involving animals were conducted as defined in Vanderbilt animal protocol number M-10-411, and according to the Animal Welfare Act and approved by the Vanderbilt Institutional Animal Care and Use Committee. Animal care/welfare and veterinary oversight was provided by the Vanderbilt Divison of Animal Care.

### Cell culture

The previously established human bladder cancer cell lines RT4 [10] and T24 [11] were maintained in McCoy's modified medium supplemented with 10% FBS. J82 [12], 5637 [13] and UMUC-3 [14] human urothelial cancer cell lines were cultured in DMEM with L-glutamine and high glucose (4.5 g/L; Mediatech) supplemented with 10% FCS (Atlanta Biologicals) and 100 units/mL penicillin/streptomycin (BioWhittaker/Cambrex). 253J–P and 253J-BV [15] cells were obtained through a material transfer agreement from the laboratory of Dr. Colin Dinney (University of Texas MD Anderson Cancer Center) and cultured in MEM (Mediatech) supplemented with 10% FCS (Atlanta Biologicals), 100 units/mL penicillin/streptomycin (BioWhittaker/Cambrex), 10 mmol/L sodium pyruvate (Mediatech), 1x nonessential amino acids (Mediatech) and 2x MEM vitamin solution (Mediatech). SCaBER cells [16] were obtained from ATCC and maintained in Minimum essential medium (Eagle) (Invitrogen) containing 10% FCS with 2 mM L-glutamine (Mediatech) and Earle's balanced salt solution (Invitrogen) adjusted to contain 1.5 g/L sodium bicarbonate, 0.1 mM non-essential amino acids, and 1.0 mM sodium pyruvate. The previously established human prostatic adenocarcinoma cell lines LNCaP [17] and PC3 [18] and the hepatocellular carcinoma cell line HepG2 [19] were maintained in RPMI 1640 supplemented with 10% FBS. All cell lines were maintained at 37°C in 5% CO_2_.

### PCR

RNA extraction was performed with RNeasy kits (Qiagen) according to manufacturer's protocol with Dnase digestion. Subsequently, cDNA was amplified according to standard protocols. PCR was performed with primer sets for human FOXA1 (fwd-CGCTTCGCACAGGGCTGGAT, rev-TGCTGACCGGGACGGAGGAG), FOXA2 (fwd-TGCCATGCACTCGGCTTCCA, rev-CCCAGGCCGGCGTTCATGTT), FOXA3 (fwd-CTGGCCGAGTGGAGCTACTA, rev-GAGGATTCAGGGTCATGTAGGA). Primer sequences used for standard PCR of uroplakin transcripts have been previously reported [20]. Primer sets for Q-RT-PCR analysis include UPK1A fwd-GGTGTGGGTGCCGCACTCTG, rev-GGTCGGTGTCCGCGCTGTAG), UPK1B (fwd-GCCCTACCGTGTGCGCAGAAA, rev-AGCAGGCCCTGGAAGCAACG), UPK2 (fwd- CTCTGCTGTCCCCAGGGGCT, rev-GGCAACCAGCAGGCTCTCCG), UPK3A (fwd-TCACTGGCACCCACGAGGTCT, rev-CGTTGAGCCCAGTGGGGTGTT), and UPK3B (fwd- CCCTGGCCCTGGACCCTATCG, rev-CCACAGGCTGGAGAAGCGCA). GAPDH (fwd-TGCACCACCAACTGCTTAGC, rev-GGCATGGACTGTGGTCATGAG) was used as reported previously [21] as an internal control for PCR reactions.

### Human tissue samples

The present study included two separate patient cohorts from Vanderbilt University Medical Center and the University of Virginia Medical Center (summarized in Table 1). All studies were performed following approval of the Internal Review Boards at each of the participating institutions. Whole tissue sections were prepared from transurethral endoscopic tumor resection samples from 18 patients with low grade stage Ta disease treated at Vanderbilt University Medical Center. Tissue samples from primary tumor samples derived from the University of Virginia patient cohort were represented on a previously described tissue microarray (TMA) [9]. Quadruplicate tissue cores (0.6 mm) of viable tumor were harvested from archived zinc-formalin-fixed tissue blocks of 167 cystectomy specimens. The original pathology slides were reviewed to record the histologic subtype and grade as well as confirm the pathologic stage of the tumors. Tumors were histologically graded on a scale from 1–4, with grade 4 as the highest grade. Of 19 patients who received neoadjuvant therapy, 6 were treated with radiation therapy, 8 with chemotherapy and the remainder with combination chemoradiotherapy. The TMA cohort included 112 urothelial carcinomas, 21 squamous cell carcinomas, 5 primary bladder adenocarcinomas, 3 small cell (high-grade neuroendocrine) carcinomas, 1 mixed urothelial and squamous carcinoma, 1 adenocarcinoma with sarcomatoid foci and 2 urothelial carcinomas with sarcomatoid foci (Table 1). An additional TMA from the University of Virginia consisted of quadruplicate tissue cores (0.6 mm) of lymph node metastases dissected from 28 patients represented in the primary tumor TMA (Table 1).

**Table 1:** Tissue sources and demographic information.

Vanderbilt University Cohort		
Gender (%)	Male	14 (77%)
	Female	4 (23%)
Mean age	67	
T stage (%)	Ta	18 (100%)
N stage		N/A
Grade		Low

University of Virginia Cohort		
Gender (%)	Male	103 (71%)
	Female	42 (29%)
Mean age	67	
T stage (%)	TaT1T2T3T4	4 (3%)6 (4%)47 (32%)64 (45%)23 (16%)
N stage (%)	N0N1 or higher	39 (60%)26 (40%)
Grade (%)	Grade 1–2Grade 3Grade 4	26 (23%)72 (63%)17 (14%)

In addition to the tumor samples for which demographic data were available as described in Table 1, archival tissue consisting of tumor and normal adjacent tissue (NAT) from 10 patients who underwent cystectomy for advanced bladder cancer at Vanderbilt University were collected for the analysis of FOXA1 and uroplakin. In addition, archival tissue consisting of 21 patients with non-keratinizing squamous metaplasia were identified. Of these, 2 patients had areas of keratinizing squamous metaplasia, and 15 patients had NAT, which served as a control.

### Immunohistochemistry and dual immunofluorescence

Immunohistochemistry was performed as previously described [22], [23]. Briefly, slides were deparaffinized, rehydrated through a series of graded alcohols and washed in double deionized water for 5 minutes. Tissues were then placed in antigen unmasking solution (Vector Labs, Burlingame, CA) and antigen retrieval was performed by microwaving samples for 20 minutes at 30% power in a 900 watt microwave oven. Slides were then cooled to room temperature, and then washed 3 times for 10 minutes in PBS (pH 7.4). For immunohistochemistry, all incubations were performed at room temperature unless otherwise stated. Endogenous peroxidase activity was blocked with the use of Peroxidase blocking reagent (Dako North America, Carpinteria, CA) for 20 minutes, after which sections were again washed in PBS 3 times for 10 minutes. Prepared slides were incubated in goat serum for 30 min to reduce non-specific antibody binding. Slides where then incubated overnight at 4°C in a humidified chamber with 1∶1000 dilutions of either goat polyclonal FOXA1 (Santa Cruz Biotechnology, Santa Cruz CA), goat polyclonal FOXA2 (Santa Cruz Biotechnology) or a pan-uroplakin antibody, AUM [24] diluted 1∶5000 in PBS. Slides were then washed 3 times for 10 min with PBS and biotinylated secondary antibody diluted in PBS was then added. Primary antibody was visualized using the Vectastain Elite ABC Peroxidase kit (Vector Labs) according to the manufacturer's protocol with DAB in substrate buffer as chromogen (Thermo Scientific, Fremont CA). For FOXA1 and FOXA2, slides were scored for the presence or absence of specific nuclear staining. The positive control for FOXA1 were wild-type murine prostate tissue, and the positive control for FOXA2 was prostatic tissue from previously described Probasin-T-antigen//Dominant active beta-catenin bigenic mice [21]. For pan-UPK, the presence or absence of cytoplasmic immuno­reactivity in tumor cells was recorded. Immunostaining of human bladder tissue was performed for the squamous epithelium marker cytokeratin 10 (1∶100; Dako, Carpinteria CA), and the basal cell marker cytokeratin 14 (1∶200; Dako). Immunofluorescence staining was performed on human bladder tissues with antibodies for Ki67 (1∶100; Dako; Carpinteria, CA; clone MIB-1) and FOXA1 (1∶100: Santa Cruz).

### Stable Cell Line Generation

Following transfection of Phoneix packaging cells, retroviral particles were filtered and purified, and viral particles were used for infection of RT4 and T24 target bladder cell lines. Following retroviral infection, RT4 cells were puromycin selected to stably express a FOXA1 targeted shRNA construct resulting in decreased FOXA1 expression (RT4-FOXA1 KD) or scrambled shRNA expressing control cells (RT4-Scr) according to manufactures instructions (Origene, Rockville, MD). Similarly, T24 cells were puromycin selected following viral infection to stably express pLPCX plasmid containing a FOXA1 insert (T24-FOXA1) or empty vector (T24-pLPCX). Data from microarray analysis described in this manuscript will be deposited in a publically available database in compliance with MIAME guidelines.

### Tissue Recombination Xenografting

All animal experiments were performed in accordance with institutional IACUC approval. Isolation of embryonic bladder mesenchyme (eBLM), preparation of tissue recombinants, and kidney capsule surgeries were performed as described previously [2]. Pregnant rats (Harlan Laboratories, Tampa FL) were sacrificed at embryonic day 16 (E16) (plug day = 0). Bladders were then microdissected from isolated embryos, and embryonic bladders were separated from the urogenital sinus at the bladder neck and the attached ureters carefully dissected. Whole bladders were then placed into calcium and magnesium-free Hanks' saline (Gibco) containing 25 mM EDTA (Sigma, St. Louis MO) for 90 min to release the bladder urothelium. The mesenchyme and urothelium were separated manually under microscopic examination, leaving the mesenchyme behind as a bladder shell. Fifty thousand RT4-Scr cells and RT4-FOXA1 KD cells were re-suspended in 50 microliters of a 3∶1 ratio of rat tail collagen and setting solution, and were plated in 10 cm dishes. Following the insertion of 1 eBLM per aliquot, tissue recombinants were placed at 37°C to promote solidification. McCoy's modified medium (Gibco) containing 10% FBS was then applied to solidified grafts and incubated overnight. The following day, two tissue recombinants were placed under the kidney capsule of the left kidney of 5 SCID mice, resulting in a total of 10 grafts. Three weeks following implantation, mice were injected with BRDU and sacrificed. Dissected kidneys containing tissue recombinants were fixed in formalin and subjected to standard processing in preparation for immunohistochemistry. Animal experiments were repeated once. Tumor volume measurements were performed by standard methods and are represented as fold tumor volume.

### Western blotting analysis

Western blotting analysis was performed as reported previously [25]. Briefly, cell lysates were prepared with Complete Lysis-M kit (Roche, Nutley NJ) as per manufacture protocol and protein concentrations were determined via standard BCA protocol (Pierce, Rockford, IL). Cell lysates (30 µg) were subjected to electrophoresis on a 10% Bis–Tris gels for 50 min at 200 V. Electrophoretic transfer to nylon reinforced nitrocellulose membranes (Osmonics, Minnetonka, MN) was performed overnight at 30 V, followed by Ponceau-S staining (Sigma) in order to verify equal protein loading and transfer. Membranes were blocked overnight in in 5% non-fat dry milk (NFDM), 0.1% Tween 20 (Sigma) in 1x Tris buffered saline. The following day, nitrocellulose membranes were incubated with goat anti-FOXA1 (Santa Cruz; 1/1000), anti E-cadherin (BD Biosciences; 1/1000) or anti beta actin (Sigma; 1/1000) followed by appropriate HRP-conjugated secondary antibody (Jackson Labs, Bar Harbor, ME) at a dilution of 1/2,000.

### Crystal violet growth assays

Cells (5,000 per well) were plated in 24 well culture dishes (Falcon) and allowed to attach overnight in McCoy's medium containing 10% FBS and 0.5 µg/ml puromycin. The following day, culture medium was aspirated, and cells were fixed in 11% glutaraldehyde (Sigma) and incubated at room temperature on an orbital shaker set at 500 cycles/min. Cells were then washed 3 times by submerging in deionized water and allowed to air dry and stained with 0.1% crystal violet dissolved in 200 mM boric acid (Sigma), pH 8.0. Following incubation for 20 minutes on an orbital shaker, excess crystal violet was then removed by washing in deionized water and allowed to air dry. Crystal violet stain was subsequently dissolved in 10% acetic acid (Sigma) and absorbance was read at 590 nm following background subtraction. Crystal violet growth assays were performed for five days in triplicate and repeated twice.

### In vitro invasion assays

*In vitro* invasion assays were performed as previously reported [26]. Falcon cell culture inserts (8 µm pores) were washed twice with McCoy's medium and subsequently coated with 20 µl of reduced growth factor Matrigel (BD Biosciences, Franklin Lakes NJ) diluted 1∶6 and allowed to solidify for 30 minutes. RT4-Scr, RT4-FOXA1 KD, T24-pLPCX, and T24-FOXA1 (1×10^5^/well) were added to individual wells in 200 µl if McCoy's medium (10%FBS, 0.5 µg/ml puromycin), and 300 ul of identical medium was added to the bottom chamber. After 24 and 48 hours incubation, medium was aspirated from the lower chamber, and invaded cells were fixed in 5% glutaraldehyde dissolved in 1x PBS for 10 min at room temperature, and cells were subsequently washed in deionized water three times. Invaded cells were stained by adding 0.5% toluidine blue (Sigma) in 2% sodium carbonate and incubating 20 min, after which toluidine dye and medium was aspirated from the upper and lower chamber, respectively. The bottom chamber was then washed three times with deionized water, and non-invaded cells were removed by wiping the inside of the upper chamber gently with a cotton swab, and invaded cells were counted under a 20x objective. Invasion assays were performed in triplicate and repeated twice.

### Statistical analysis

Associations between nuclear FOXA1 and FOXA2 staining and clinicopathologic parameters were assessed using standard univariate methods. FOXA1 and FOXA2 expression status was summarized by tumor stage, nodal status, histologic type and grade. χ2 tests of association were performed between dichotomized expression values and each pathologic parameter. For FOXA1 expression, logistic regression was used to test the predictor “tumor stage” as an ordinal variable with five categories (Ta, T1, T2, T3 and T4). Tests of association were performed both on the entire data set and the subset of TCC samples only. Statistical analysis was performed in SAS 9.2. All tests were assessed at α = 0.05. Statistical evaluation of differences in tumor volume between RT4-Scrambled and RT4-FOXA1 KD were performed by standard univariate methods, with p<0.05 considered significant.

## Results

**Figure 1:**
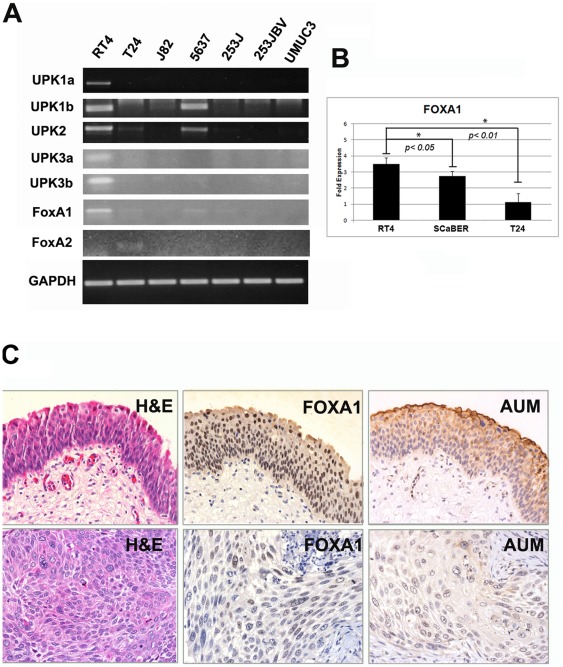
Analysis of FOXA1 and uroplakin expression in vitro and in human bladder tissue. A: A panel of commonly used urothelial cell lines was screened via traditional RT-PCR for the presence of FOXA1, FOXA2, and FOXA3 transcripts, as well as members of the uroplakin (UPK) family. HepG2 cells, which express each member of the FOXA subfamily, were used as positive controls (data not shown). RT4 cells exhibited robust expression of FOXA1 and as previously reported, UPK family members [20]. FOXA2 expression was detected in T24 cells, but was not correlated with UPK family member expression. FOXA3 was not detected in any tested cell line (data not shown). B:Q-RT-PCR analysis shows FOXA1 expression in the SCaBER cell line derived from a primary SCC and in T24 is significantly lower compared to RT4. C∶Decreased FOXA1 expression is associated with decreased UPK expression in human tissue. Archival normal adjacent tissue (top panel) and muscle invasive bladder tumor was immunostained with a pan-UPK antibody, AUM [24] as well as an antibody directed against FOXA1. Out of 10 samples with FOXA1 positive NAT, and FOXA1 negative associated tumor, 4 tumors exhibited negative AUM staining.

FOXA1 and FOXA2 expression is restricted to specific bladder cancer cell lines:

In studies of urinary bladder development, nuclear FOXA1 expression was found to be restricted to the urothelial compartment and was maintained in adult urothelium [27]. In contrast, *in situ* hybridization analyses indicated expression of FOXA2 during early embryonic development [28], while no expression was evident in later stages of urothelial development or in the urothelium of adult bladders [27]. As an initial step in characterizing the expression of FOXA family members in bladder cancer, we performed RT-PCR on the commonly used human bladder cancer cell lines RT4, T24, J82, 5637, 253J, 253J-BV, UMUC3 and SCaBER. Interestingly, only the RT4 cell line, which was originally propagated from a well differentiated tumor [10], exhibited high FOXA1 expression. In addition, expression of FOXA1 was associated with the presence of UPK transcripts (Fig 1A), of which UPK2 is used as a urothelial differentiation marker. 5637 cells also co-expressed low levels of FOXA1 and UPK mRNA. T24 cells expressed very low levels of FOXA1 and UPK2, but also showed evidence of FOXA2 expression (Fig. 1A). Quantitative RT-PCR analysis of SCaBER cells established from a primary SCC tumor showed that FOXA1 expression was significantly lower in these when compared to RT4 (Fig. 1B). None of the cell lines examined expressed FOXA3, a third related member of the FOXA family (data not shown). As FOXA1 expression was associated with UPK in RT4 and 5637 cells, we investigated the expression of these markers in human bladder cancer specimens. Out of 10 samples with FOXA1 positive NAT, and FOXA1 negative associated tumor, 4 tumors exhibited negative AUM staining, while 6 tumors retained AUM staining (Fig. 1C). Taken together, these results indicate that loss of FOXA1 expression is associated with decreased or absent UPK expression in widely used human bladder cancer cell lines and in a subset of human bladder tumor tissue.

### FOXA1 knockdown in RT4 cells increases uroplakin expression

**Figure 2:**
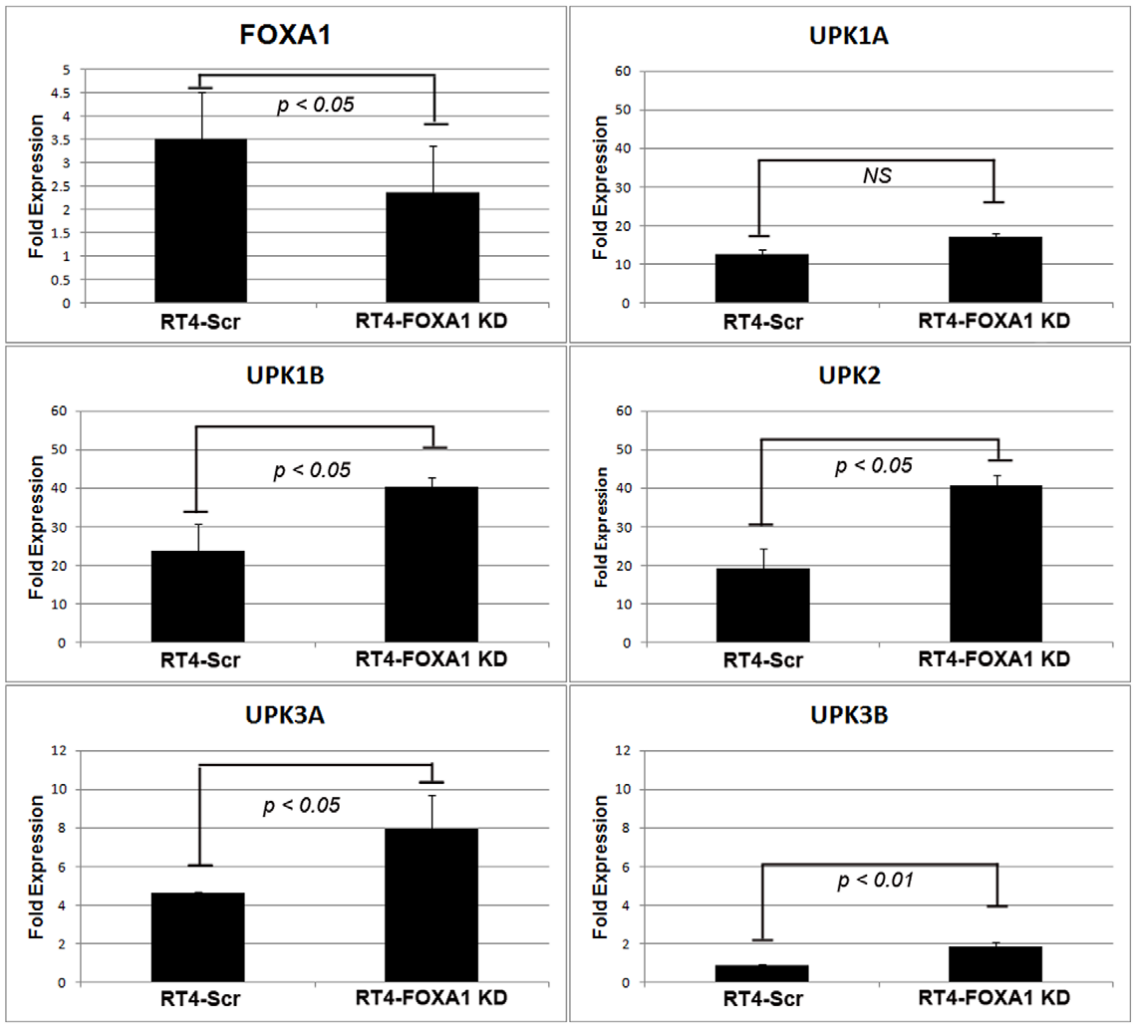
Silencing of FOXA1 in RT4 cells results in increased expression of UPK family members. A: Stable knockdown of FOXA1 in human RT4 bladder cancer cells (See Figure 6 for FOXA1 protein levels following knockdown in RT4). B: No significant changes in UPK1A were detected following FOXA1 KD. (C-F): mRNA levels of UPK1B, UPK2, UPK3A, and UPK3B were significantly increased following FOXA1 knockdown.

The uroplakin (UPK) family of genes in humans consists of UPK1A, UPK1B, UPK2, UPK3A and UPK3B [29]. Although decreased FOXA1 expression appeared to be correlated with diminished UPK expression in commonly used bladder cancer cell lines, and in a minority of human cystectomy samples (Fig 1), the AUM antibody used for these studies recognizes all members of the uroplakin family [30]. Furthermore, it was previously reported that knockdown of FOXA1 resulted in both decreases and increases in the expression of UPK family members [5]. Therefore, it was unclear if altered FOXA1 expression resulted in changes of individual uroplakin family members. As RT4 cells express FOXA1 and each member of the UPK family (Fig 1), we used an shRNA approach to engineer stable RT4 cells with decreased FOXA1 expression to determine the impact of FOXA1 silencing on UPK expression (Fig 2). Interestingly, microarray studies performed on RT4-Scr and RT4-FOXA1 KD indicated FOXA1 KD was associated with increased UPK family member expression. Quantitative RT-PCR studies of RT4-Scr and RT4-FOXA1 KD cells validated this observation, showing that FOXA1 KD resulted in significant increases in the expression of UPK1B, UPK2, UPK3A, and UPK3B (Fig 2C–F). Thus, although FOXA1 and UPK are lost in the majority of cell lines established from high grade, MI tumors and in a subset of human cystectomy samples, FOXA1 KD in RT4 cells results in significantly increased UPK family member expression *in vitro*.

### Loss of FOXA1 expression is associated with advanced tumor stage and high histologic grade

**Figure 3:**
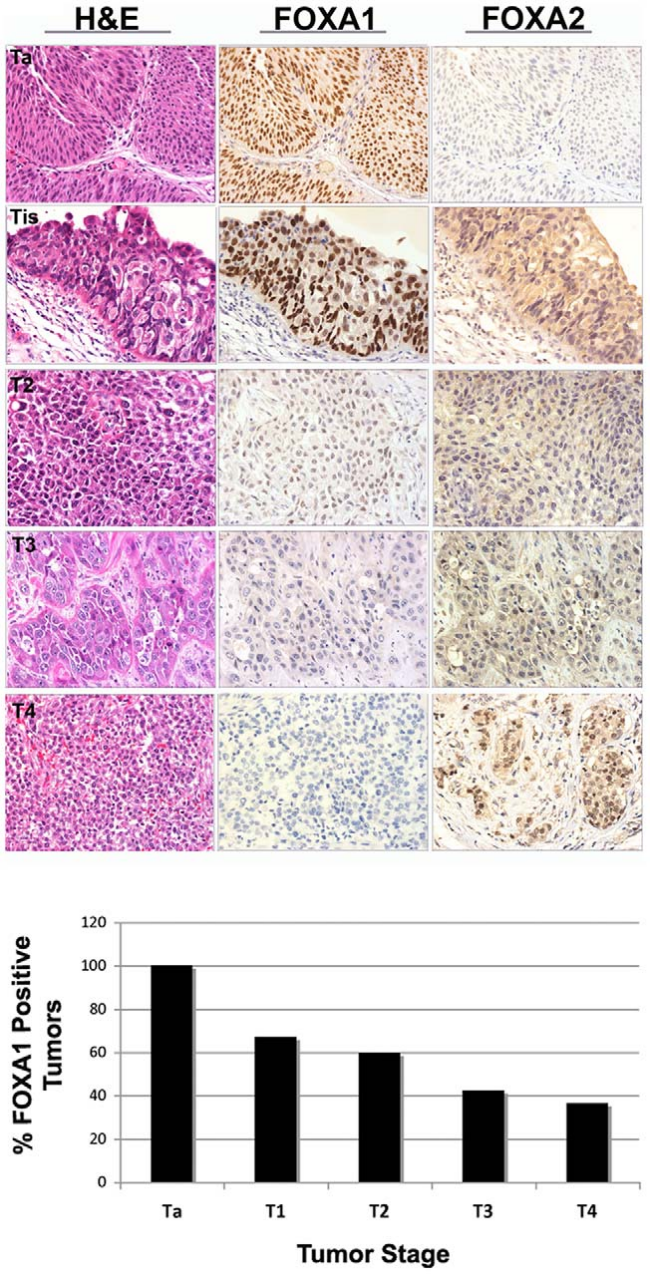
FOXA1 expression is lost in most in high grade, advanced stage muscle-invasive bladder cancers. AJCC stage Ta, T1, T2, T3 and T4 bladder tumors were immunostained for FOXA1 and FOXA2. Representative cases are illustrated in top panels. Tis area is depicted in a patient diagnosed with AJCC T1 stage bladder tumor. Association between loss of FOXA1 expression and increasing stage was confirmed by logistic regression (bottom panel, p<0.001). A small subset of invasive tumors exhibited nuclear expression of FOXA2.

To determine the extent of FOXA1 and FOXA2 expression in various stages of bladder cancer, we performed immunohistochemical analysis on human tumor samples (Fig. 3). The prevalence of FOXA1 expression decreased with increasing tumor stage (Table 2). FOXA1 was uniformly expressed in stage Ta bladder tumors, compared to only 67% of stage T1 tumors, 59% of stage T2 cancers, 42% of stage T3 tumors, and 34% stage T4 neoplasms. Logistic regression analysis demonstrated that loss of FOXA1 expression was significantly associated with increasing tumor stage (p<0.001). Loss of FOXA1 expression also occurred in tumors of higher histologic grade (p<0.001; Table 2). FOXA1 was also negative more often in tumors from female patients. However, this association failed to reach statistical significance following normalization to tumor stage and grade (p = 0.096). FOXA2 was not detected in Ta stage tumors and was present only in 12% of higher stage cancers. There were no significant associations between FOXA2 expression and tumor stage or histologic grade.

**Table 2:** Association of FOXA1 Staining with gender, tumor stage, grade, and nodal status.

		Total	FOXA1+ (%)	FOXA1− (%)	P value
**Gender**	MaleFemale	10342	56 (54%)15 (36%)	47 (45%)27 (64%)	p = 0.096
**Tumor Stage**	Ta, T1T2-T4	29166	27 (93%)77 (46%)	2 (7%)89 (54%)*	p<0.001*
**Tumor Grade**	G1-2G3G4	267217	20 (77%)39 (54%)2 (11%)	6 (23%)33 (46%)15 (88%)*	p<0.001*
**Nodal Status**	N0N1 or greater	3926	19 (49%)13 (50%)	20 (51%)13 (50%)	p>0.05

### FOXA1 expression is significantly reduced in keratinizing squamous metaplasia and squamous cell carcinomas of the urinary bladder

**Figure 4:**
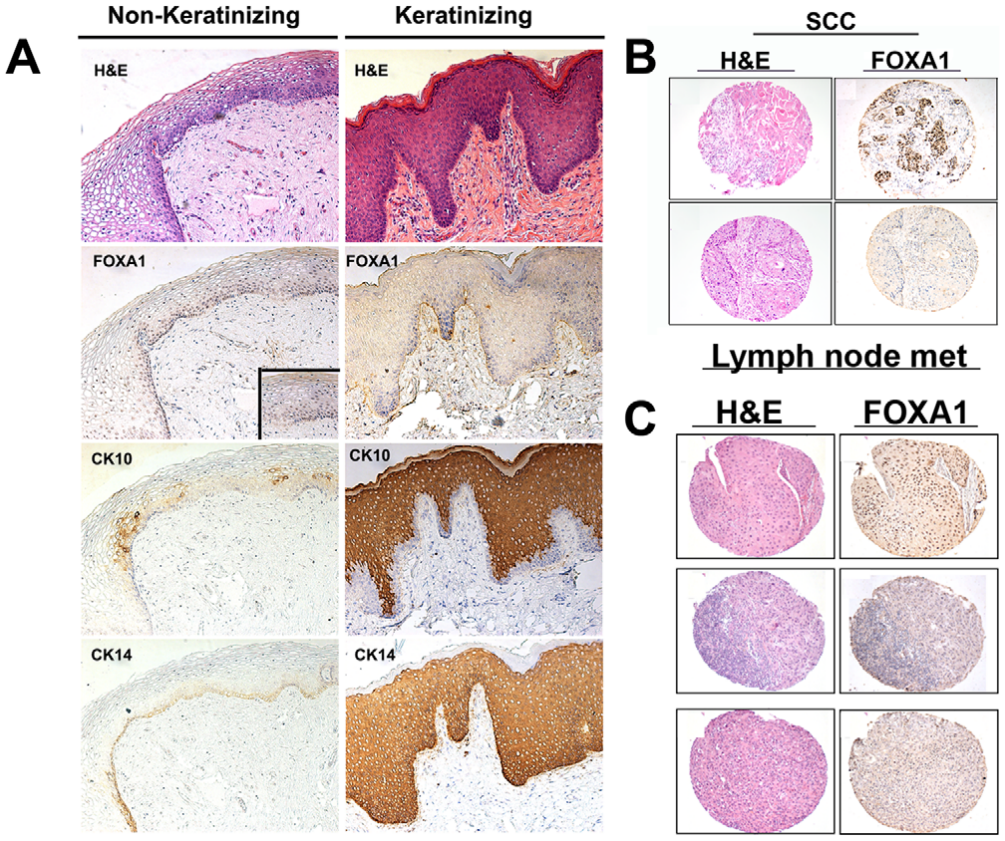
FOXA1 expression is absent in keratinizing squamous metaplasia and squamous cell carcinoma (SCC) of the urinary bladder. (A) H&E and immunostaining of non-keratinizing (left panel) and keratinizing (right panel) squamous metaplasia for FOXA1, the squamous cell marker cytokeratin 10, and the basal cell marker keratin 14. Inset on left panel shows positive FOXA1 staining at high magnification. (B) H&E (left panel) of FOXA1-positive (top) and FOXA1-negative (bottom) samples of human SCC of the urinary bladder are depicted. Most cases (81%) of bladder SCC showed loss of FOXA1 expression. (C) FOXA1 expression is lost in lymph node metastases of some patients with SCC: H&E (left panel) of FOXA1-positive UCC (top) and FOXA1-negative SCC (middle and bottom) metastatic lymph node samples isolated from bladder cancer patients are depicted.

The majority of bladder cancers are histologically identified as UCC. SCC is the next most common histological variant of bladder cancer. A recognized precursor of SCC is the development of keratinizing squamous metaplasia (KSM). KSM is distinct from non-KSM, which is relatively common and benign. As the prognosis of patients with nonbilharzial SCC of the urinary bladder is especially grave [31], we compared FOXA1 expression in KSM, non-KSM and SCC. Histological analysis revealed that while FOXA1 was expressed in non-KSM (Fig. 4A, inset, left panel), and overlapped with expression of CK14 positive basal cells, FOXA1 was not expressed in KSM (Fig. 4A) or most of SCC (Fig. 4B). FOXA1 expression was lost in 81% of SCC samples (Fig. 4B) compared to 40% of UCC of the bladder (Fisher's exact test, p<0.001) (Table 3). While the majority of SCC is diagnosed at a relatively advanced tumor stage, the association between SCC and FOXA1 loss remained significant (p = 0.0043) even after adjusting for tumor stage. Furthermore, FOXA1 staining of a TMA consisting of metastatic tumor deposits from positive lymph nodes dissected from 28 patients represented in the primary tumor TMA (Fig. 4C) revealed a further association between FOXA1 loss and SCC. Out of the original 28 cases, sufficient tissue for analysis remained in only 22 samples. Five cases of lymph node metastases (23%) were negative for FOXA1 expression. Three out of 5 FOXA1-negative lymph nodes (60%) were dissected from patients diagnosed with primary SCC. Of these, 2 (one stage T4 and one stage T2) were matched to FOXA1-negative primary tumors, one was matched to a stage T3 primary tumor with loss of FOXA1 expression. The remaining 2 FOXA1-negative lymph node metastases were dissected from patients diagnosed with stage T3 TCC and small cell carcinoma, both of which were negative for FOXA1 expression.

**Table 3:** Histological subtype and FOXA1 status.

	Total	FOXA1+ (%)	FOXA1− (%)	p value
SCC	21	4 (19%)	17 (81%)*	*p = 0.043
TCC	130	78 (60%)	52 (40%)	

### FOXA1-negative urothelium is proliferative and FOXA1 knock-down results in increased tumor proliferation and decreases in E-cadherin expression

**Figure 5:**
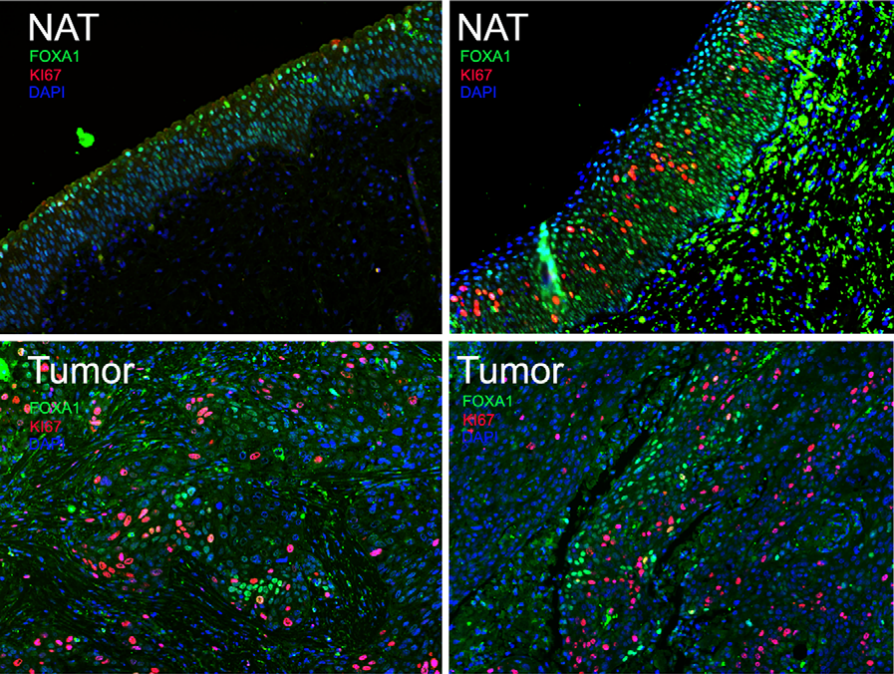
FOXA1 negative tumor cells are proliferative. Dual immunofluorescence of radical cystectomy patient samples depicting co-localization of FOXA1 (green) and proliferation marker Ki67 (red). Left panel is normal adjacent tissue (NAT, top left) and matched tumor (bottom left) from a patient with UCC, while right panels depict normal adjacent tissue (NAT, top right) and squamous cell carcinoma (bottom right). Note that while NAT from UCC depicted in left panel expresses FOXA1 and is Ki67 negative, NAT from patient with SCC displays a subpopulation of FOXA1-negative, Ki67-positive cells. Both tumors (bottom panel) show a subpopulation of cells exhibiting mutually exclusive expression of FOXA1 and Ki67.

**Figure 6:**
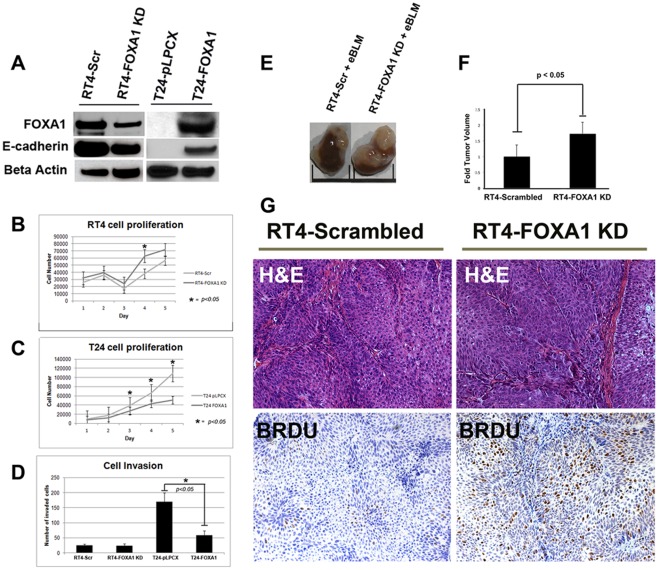
Alterations in FOXA1 expression in RT4 and T24 cells results in changes in E-cadherin expression and cell behavior. (A) Generation of RT4-Scrambled and RT4-FOXA1 KD cells, as well as T24-pLPCX (empty vector) and T24-FOXA1 overexpressing cells: FOXA1 knockdown in RT4 cells resulted in decreased E-cadherin expression (A) and significantly increased cell proliferation at day 4 (B). Overexpression of FOXA1 in T24 cells resulted in increased E-cadherin expression (A), and significantly decreased cell proliferation (C). While manipulation of FOXA1 had no impact on *in vitro* invasion of RT4 cells (D), overexpression of FOXA1 in T24 cells significantly decreased cell invasion (D). RT4 cells stably expressing scrambled construct (RT4-Scrambled) or FOXA1-specific shRNA (RT4-FOXA1 KD) were recombined with embryonic bladder mesenchyme (eBLM) isolated from embryonic-16 day old rats and inserted under the kidney capsule of immunocompromised mice. After three weeks, host mice were injected with BrdU and sacrificed. (E and F) Tumor volume was significantly increased in FOXA1-KD RT4 cells. (G) H&E (top panel) and BRDU staining (bottom panel) of RT4-Scrambled and RT4-FOXA1 KD cells. H&E staining shows presence of fibromuscular stroma following recombination with both cell lines. RT4-FOXA1 KD cells showed increased incorporation of BrdU (G), indicating FOXA1 knock down results in increased bladder cancer cell proliferation.

In order to determine the proliferative potential of intra-tumoral FOXA1 negative urothelium, we performed dual-immunofluorescence using antibodies for FOXA1 and the proliferation marker Ki67 (Figure 5). Interestingly, we found that the presence of positive FOXA1 staining and Ki67 was relatively exclusive. We compared RT4-FOXA1 KD cells to control RT4-Scr cells to determine the impact of altered FOXA1 expression levels on bladder cell *in vitro* growth. Additionally, we established T24 cells stably over expressing FOXA1 to determine the impact of restoring FOXA1 expression on cell behavior (Fig. 6). Microarray analysis utilizing Affymetrix Gene Titan chips was subsequently performed to identify FOXA1 target genes potentially responsible for any altered behavior of RT4 and T24 cells. Loss of E-cadherin expression is implicated in bladder cancer progression, and decreased FOXA activation of E-cadherin expression is implicated in pancreatic tumor progression [32]. Microarray analysis indicated overexpression of FOXA1 in T24 cells resulted in increased E-cadherin expression. Importantly, western blotting analysis of both RT4 and T24 based cell lines verified the correlation between altered FOXA1 and E-cadherin expression (Fig. 6A). We next performed crystal violet growth assays to identify a potential role for FOXA1 expression in the regulation of bladder cancer cell proliferation. RT4-FOXA1 KD cells exhibited significant increases in cell number at day 4 (Fig. 6B). While there was a trend towards increased RT4-FOXA1 KD cell growth at day 5, the difference was not statistically significant (Fig. 6B). Overexpression of FOXA1 in T24 cells significantly decreased T24 cell proliferation at days 3, 4, and 5. These results indicate that alterations in FOXA1 expression influence bladder cancer cell proliferation. As loss of FOXA1 expression was associated with increased tumor stage, we additionally performed *in vitro* invasion assays to determine the impact of altered FOXA1 expression in RT4 and T24 cells invasion. Control RT4-Scr cells were minimally invasive after 24 hours of incubation (Fig. 6D), and failed to exhibit significant invasion even after 48 hours (data not shown). Furthermore, FOXA1 knockdown failed to increase RT4 *in vitro* cell invasion following 24 and 48 hours of incubation. In contrast, overexpression of FOXA1 in T24 cells significantly decreased cell invasion at 24 hours (Fig. 6D). Taken together, these *in vitro* studies suggest alterations in FOXA1 expression have important implications for the growth and invasion of bladder cell lines. In an effort to determine the impact of reduced FOXA1 expression on RT4 tumorigenicity, we performed tissue recombination xenografting experiments with RT4-FOXA1 KD cells (Figure 6E, F, and G). RT4-FOXA1 KD and RT4-Scr cells were recombined with embryonic bladder mesenchyme (eBLM) and grafted under the kidney capsule of immune compromised mice as described in materials and methods. In agreement with *in vitro* findings, FOXA1 knockdown resulted in significantly increased *in vivo* tumor volume (Figs. 6E and F). Histologically, both control and FOXA1 knockdown RT4 recombinants resulted in the formation of relatively well-differentiated tumors complete with papillary structures and associated fibromuscular stroma (see H&E, Fig. 6G). In agreement with *in vitro* invasion assays, FOXA1 knockdown did not promote invasion of RT4 cells. However, the BrdU labeling index was increased in FOXA1 knockdown recombinants compared to control (Fig. 6G, bottom panel).

## Discussion

Despite clinical intervention, Surveillance, Epidemiology and End Results (SEER) data collected between 1998 and 2001 indicates the five-year survival rate for patients treated for stage T2 bladder cancer is approximately 63%, while the five-year survival rate for patients with stage T3 and T4 is 46% and 15%, respectively. Therefore, a high priority for bladder cancer research is to identify the genes that contribute to the biological processes underlying tumor progression and metastasis. In this study, we provide the first evidence linking alterations in FOXA1 expression to advanced tumor stage and high histologic grade. In addition, we report the loss of FOXA1 expression in KSM and in SCC of the urinary bladder, and show that reduced FOXA1 expression promotes RT4 xenograft proliferation.

Clinical and transgenic mouse studies have repeatedly implicated two independent molecular pathways instrumental in the development of urothelial neoplasia [33]. Low grade, Ta stage non-invasive bladder tumors are associated with activating mutations in components of the FGFR3 receptor tyrosine kinase pathway [34]. While these tumors often recur locally, they less often progress to MIBC. Conversely, the initiating lesion for the development of MIBC is chiefly *carcinoma in situ* (CIS). While Ta tumors and urothelial CIS are both associated with loss of heterozygosity (LOH) of chromosome 9 p and 9 q, progression to MIBC occurs with inactivating mutations in p53 and the Rb family of cell cycle repressors, as well as LOH on chromosomes 8 p, 11 p, 13 q, and 14 q [33], [34]. The gene for FOXA1 is located on chromosome 14 q, and we found that FOXA1 expression was uniformly present in Ta stage tumors, but was lost in Stage T2 or higher bladder cancers. One potential source of bias is that the majority (18 out of 22) of Ta stage tumors were acquired from Vanderbilt. While it will be important for other research groups to validate our finding that FOXA1 expression is uniformly positive in Ta stage tumors, it is important to point out that the significant association between loss of FOXA1 and increasing tumor grade (p<0.001) and stage (Ta, T1, T2 vs. T3, T4) remains for both UCC (p = 0.009) and SCC (p = 0.002) when the University of Virginia cohort is analyzed alone. Therefore, our results place FOXA1 loss in a molecular context that is associated with MIBC. Perhaps initial molecular “hits” resulting in the inactivation of one allele of FOXA1 occur in urothelial dysplasia/CIS, with subsequent loss of both alleles or down-regulation of the second allele occurring in more advanced MIBC. It is important to note that logistic regression analysis of all histologic tumor types and the separate subset of UCC tumors alone revealed a significant association between loss of FOXA1 and increasing tumor stage. This indicates that although approximately 80% of T2–T4 SCCs are negative for FOXA1, SCC are not the only cancers that show FOXA1 loss and advanced tumor stage. These results would seem to implicate FOXA1 in the neoplastic progression of bladder cancer, consistent with its potential role in the maintenance of a differentiated urothelial phenotype. The loss of FOXA1 expression in most SCCs also suggests that this protein is a key regulator of the normal urothelial cell phenotype. Our finding that FOXA1 expression was lost only in a minority of lymph node metastases was surprising, as we expected the majority of lymph node metastases to exhibit negative FOXA1 staining. It has been recently reported that cytokeratin 20 positive cells can be detected in the bone marrow aspirates of cystectomy patients, regardless of primary tumor stage [35]. This observation and similar ones in the field of breast and prostate cancer may be explained by the theory of parallel progression of metastasis [36]. This theory suggests that the metastatic cascade of tumor cell dissemination to distal tissues occurs much earlier than commonly thought. The fact that a minority of lymph node metastases were negative for FOXA1 may indicate that cancer dissemination to lymph nodes occurs prior to loss of FOXA1 within the primary tumor. Alternatively, FOXA1 loss may not be important for lymph node metastases, or microenviornmental cues within the lymph node may somehow result in reactivation of FOXA1 expression. Still, our analysis shows approximately one quarter of lymph node metastases are negative for FOXA1. Most importantly, 3 out of 5 FOXA1-negative lymph nodes (60%) were dissected from patients diagnosed with primary SCC, suggesting a particular link between FOXA1 loss and lymph node metastases in patients with SCC. The phenotypic differences between FOXA1 positive and negative metastases are unknown, and it is possible that FOXA1 status in metastatic tissue correlates with clinical outcome. Therefore, future work will be directed toward evaluating this important issue.

After adjusting for tumor stage, our results show a trend towards a significant association between loss of FOXA1 expression and female sex (p = 0.096). Should future studies reveal a significant relationship between FOXA1 loss and gender, the fact that FOXA1 has been described as a “master” of steroid receptor function in malignancy (reviewed in [37]) indicates further studies of the relationship between FOXA1 expression and gender in bladder cancer are warranted. Our laboratory reported the direct interaction of FOXA1 with the androgen receptor (AR) and showed the importance of FOXA1 during normal prostate development [23], [38] which is a androgen-regulated process. Subsequently, it was reported that FOXA1 expression was essential for estrogen receptor (ER) function and mammary gland development [39], [40]. While incidence of bladder canc-er is highest among men, tumor recurrence and disease-specific mortality is higher among women [41], [42]. It will be important to correlate FOXA1 expression with disease specific outcome in future studies, and to determine the functional significance of FOXA1 expression for AR and ER activity, as these receptors have been implicated in the molecular pathogenesis of bladder tumors.

While this is the first study of FOXA1 expression in bladder cancer, there is extensive evidence supporting its role in urothelial differentiation. We previously demonstrated that recombination of murine embryonic stem cells (ESCs) with murine bladder mesenchyme induces ESC differentiation into tissue histologically similar to bladder mucosa. In this model, UPK expression (a marker of urothelial differentiation) is associated with loss of FOXA2 expression and maintenance of FOXA1 expression [2], [3]. Recently it was shown that treatment of mouse ESCs on a collagen matrix with all-trans-retinoic acid resulted in the downregulation of the pluripotency marker Pou5f1 (Oct3/4) and parallel upregulation of markers of superficial or “umbrella” cells, including several Upk family members (Upk1a, Upk1b, Upk2, Upk3a and Upk3b) and Keratin 20 [4]. Induction of these markers of terminal urothelial differentiation was accompanied by increased expression of Foxa1 and Foxa2, potentially through the direct regulation of retinoic acid receptors. Also, It has been reported that treatment of normal human urothelial (NHU) cell cultures with the PPAR-γ agonist troglitazone and inhibition of EGFR signaling causes in increased FOXA1 expression and subsequent binding of FOXA1 to UPK promoters that results in increased transcription [5]. However, our immunostaining analysis showed that out of 10 FOXA1 negative tumors with FOXA1 positive NAT, 6 retained AUM staining, indicating the retention of UPK family member expression. This is consistent with the observation that up to 80% of primary tumors retain the expression of at least one UPK gene [20]. Thus, while a subset of FOXA1 negative tumors lose UPK expression, loss of FOXA1 and UPK staining are not mutually inclusive. This conclusion is supported by our finding that stable knockdown of FOXA1 resulted in significant increases in the expression of UPK1B, UPK2, UPK3A, and UPK3B (Fig. 2). Our finding that reduced FOXA1 expression results in increased UPK family member expression is in agreement with those of Varley et. al. [5], which showed FOXA1 silencing in NHU cells resulted in decreases in UPK1A, UPK2, and UPK3A, and increases in the expression of UPK1B and UPK3B.

While the UPK family of genes is expressed in the epithelium of a relatively limited number of tissues, the mechanisms responsible for the tissue-specific nature of UPK expression are largely unknown. The combinatorial control theory of gene expression [43] suggests the combination of different transcription factors (and other genetic and epigenetic events), which are themselves expressed in a temporally and spatially regulated manner, contributes to the expression of genes in a tissue-specific manner. While no one transcription factor can be responsible for the regulation of a given promoter, our results, and those of other investigators show that FOXA1 is an important modulator of UPK family member expression. However, it is important to note that promoter regulation is achieved through the coordinated binding of several transcription factors to *cis* regulatory binding sites, and UPK promoters are no exception. For example, steroid hormone binding proteins such as members of the PPAR family have been shown to be important for UPK promoter regulation [44]. Furthermore, the transcription factor IRF-1 was recently shown to play an important part in the regulation of UPK promoter activity [5]. This would explain why although FOXA1 is expressed in a variety of tissues, including the liver and prostate, specific UPK proteins are not. Moreover, although FOXA1 is seemingly expressed throughout the various layers of the urothelium, UPK family members are relatively restricted to the most luminal superficial or “umbrella” cell layer. This is probably true because umbrella cells express transcription factors (and other important regulatory proteins) that normally cooperate with FOXA1 to restrict and regulate UPK promoter activity specifically in umbrella cells. It is unclear why both FOXA1 and UPK proteins are absent in the majority of cell lines examined in this study, while only a subset of human tumors exhibit absence of both FOXA1 and AUM (UPK) expression (Fig. 1). However, our observation that FOXA1 knockdown in RT4 cells resulted in increased UPK expression (Fig. 2) may provide an explanation. FOXA1 expression may act to maintain UPK family member expression at a relative steady state, and diminished FOXA1 expression may trigger an as yet unidentified compensatory mechanism in RT4 cells to elevate UPK family member expression. Compensatory mechanisms may include an increase in the expression of IRF-1, PPAR isoforms, retinoic acid receptor family members, and/or other unidentified factors. We have performed microarray analysis on our engineered RT4 lines, and as this information is available to other investigators, it may help in the identification of such factors. The possibility that FOXA1 knockdown triggers a compensatory mechanism, which acts to increase UPK expression, is supported by the observation that even though FOXA binding sites were identified in UK1B, FOXA1 KD in immortalized urothelium resulted in increased expression of UPK1B [5]. Alternatively, the net effect of FOXA1 expression may be to repress UPK levels, or this observation may be more reflective of the limitations of the RT4 cell line. In summary, the role of FOXA1 in the regulation of UPK proteins is complex, and more effort should be invested in lines of investigation directed at uncovering the primary determinants of urothelial-specific gene expression, as increased understanding of the mechanisms that regulate UPK expression can reveal important insights into the mechanisms responsible for normal bladder organogenesis and urothelial differentiation.

In addition to loss of FOXA1 expression in a subset of UCC, FOXA1 was negative in KSM and ∼80% of SCC. In the western world, KSM and SCC of the urinary bladder most often occurs in patients who suffer from chronic bladder inflammation, often associated with long term use of catheters to facilitate bladder emptying in the setting of chronic bladder outlet obstruction or in patients with paraplegia or chronic urinary tract infections [31], [45]. Interestingly, deficiency of vitamin A, the precursor of retinoic acid, has also been shown to lead to KSM in a number of different epithelial types [46], [47], [48]. Diet-induced vitamin A deficiency has been shown to result in KSM within the urothelium, apparently initiated in a subpopulation of progenitor cells in the proximal urethra and extending to trigone-associated urothelium and restricted regions of the bladder dome [49]. The observation that FOXA1 expression is induced by *all trans*-retinoic acid treatment in ESC [4], as well as the demonstration that FOXA1 expression is regulated by binding of the retinoic acid/vitamin A receptor to the *cis* regulatory region of the FOXA1 gene [50] implicates down regulation of FOXA1 during development of KSM. These findings may also provide an explanation for the loss of FOXA1 observed in our analysis of KSM and SCC. The connection between vitamin A, FOXA1 and squamous differentiation requires further investigation.

Our observation that expression of FOXA1 and the proliferation marker Ki67 in human bladder cancer specimens is largely mutually exclusive suggests alterations in FOXA1 expression influence bladder cancer cell proliferation. This observation is supported by the fact that silencing of FOXA1 in RT4 human bladder cancer cells enhanced *in vitro* and *in vivo* RT4 proliferation and significantly increased tumor volume. In further support of this conclusion, overexpression of FOXA1 in T24 bladder cancer cells significantly decreased *in vitro* proliferation. However, while FOXA1 silencing resulted in increased *in vivo* tumor proliferation, this did not appear to result in increased invasion in our tissue recombination experiments. This is in contrast to our finding that forced expression of FOXA1 decreased the *in vitro* invasiveness of T24 cells. A major genetic difference between the established RT4 and T24 cell lines is the status of p 53 and PTEN expression. While RT4 cells are wild-type for p 53 and PTEN, T24 cells are p 53 null and PTEN mutant. The fact that FOXA1 knockdown had little influence on RT4 invasiveness, while FOXA1 overexpression slowed T24 cell invasion *in vitro* may suggest that loss of FOXA1 expression may cooperate with inactivation of p 53 and or PTEN to promote aggressive behavior of bladder cancer cells. While this needs further experimental verification, such a suggestion is supported by the recent report that dual inactivation of p 53 and PTEN was required to drive RT4 invasion in a tissue recombination model [51]. In addition, microarray analysis indicated FOXA1 overexpression in T24 cells increased expression of E-cadherin. These findings were verified by western blotting analysis (Fig. 6). Recently, FOXA binding sites were identified in the E-cadherin promoter, and diminished FOXA1 expression was shown to result in decreased E-cadherin expression in poorly differentiated pancreatic ductal adenocarcinoma [32]. Loss of E-Cadherin expression has been repeatedly implicated in the aggressive behavior of bladder cancer [52], [53], [54], and future work is planned to investigate the link between FOXA1 and E-cadherin in bladder cancer.

The tissue recombination xenografting system is unique because it combines the strength of sub-cutaneous xenografting (relative cost effectiveness) and orthotopic xenografting (provision of stromal component) to foster increased understanding regarding the influence of tissue microenvironment. This approach has been used by other investigators to extensively study the role of stromal-epithelial interactions in urogenital development [2], [55], [56], prostate cancer [57], [58], and more recently in bladder cancer studies [51]. This is because an important defining strength of the tissue recombination model is the ability to use genetically manipulated eBLM derived from transgenic mice for tissue recombination, which can influence bladder epithelial cell growth [59], [60], [61]. While the focus of the present research was on the role of FOXA1 in BLCa cells, tissue recombination xenografting enables the incorporation of bladder mesenchyme associated determinants of disease in future studies [62], [63], [64]. Moreover, future studies exploring the cooperation between epithelial factors (such as FOXA1 loss), and stromal contributors to disease pathogenesis can be studied in this system. Therefore, as our understanding of the role of the tumor microenvironment in bladder cancer evolves, isolation of eBLM from transgenic mice, and the application of approaches used in the study of prostate differentiation and tumor progression through the isolation of transgenic urogenital mesenchyme [65], [66] will allow the design of novel experiments regarding BLCa tumor initiation and progression.

In conclusion, by showing alterations in FOXA family member expression, this work serves as the foundation for future efforts to elucidate the role of FOXA family members in the development of MIBC. Our findings both support previous reports suggesting an important role for FOXA family members in normal urothelial differentiation and suggest that gene expression networks controlled by FOXA family members may be implicated in the malignant progression of urothelial neoplasms.
